# The Hospital Nursing Supplement

**Published:** 1894-06-02

**Authors:** 


"The Hospital" Nursing Mirror.

Being the Extra Nursing Supplement of "The Hospital" Newspaper.

[Contributions for this Supplement should be addressed to the Editor, The Hospital, 428, Strand, London, W.C., and should have the word "Nursing" plainly written in left-hand top corner of the envelope.]

### News from the Nursing World.

### THE QUEEN AND THE NURSES.

A pleasant feature of the Queen's visit to Manchester was the kindly courtesy extended on that occasion to nurses from a distance, who were all accommodated with places in the Grand Stand in front of the Royal Infirmary, and hospitably entertained by the Matron. They not only saw the Queen well, but were favoured with a gracious recognition from Her Majesty. The nurses were shown over the infirmary building, and gratefully acknowledged their indebtedness to their kindly reception.

### NOT GOOD ENOUGH FOR THE POOR.

"But they are to be supervised by trained nurses," is the plausible answer constantly given to us when we call attention to the obvious impropriety of encouraging women with three months' training to adopt the title and undertake the duties of professional nurses. Experience having proved that a full course of hospital instruction, lasting at least two years, needs supplementing by twelve months' training in district nursing, we much regret the present tendency to lower the standard in certain rural districts. At the recent meeting of the Provisional Executive Committee of the Essex County Cottage Nursing Association the expression "trained" was constantly used, and evidently foreshadowed the future when every person who has had three months maternity or workhouse infirmary experience will be regarded by the poor amongst whom she is placed as a trustworthy all-round nurse. The article on "District Nurses," published on May 12th in our columns, was such an excellent summary of the advantages of the fully-trained nurse that we commend it to the notice of everyone about to organise schemes for the nursing of the sick poor. It is time that we banished for ever the inhuman theory that the life of the cottager is of so much less value than that of his well-to-do neighbour that it can be entrusted to a "respectable" partially-trained woman such as the rich man's friends would not suffer to even approach him.

### DRAINED ON THE PAVEMENTS.

The question of the infection of milk has been widely discussed in connection with an outbreak of typhoid fever in South Lambeth, but there is a wholesale pollution of milk cans going on daily before the eyes of every intelligent nurse in the poorer districts of the metropolis. At certain hours of the day outside the dairy shops boys are to be seen "cleaning" the tin receptacles. But the process is only a nominal one, for a small quantity of water and an indifferent scrubbing brush are totally inadequate for the purpose, and the subsequent reversal of the vessels "to drain" on the edge of the dirty pavement and foul gutter is the appalling conclusion of this cleansing of cans destined to receive a fresh supply of milk to be retailed for human consumption. Every nurse should call attention to such terrible evils, and advise her private or district patients to avoid dealing with any dairy where these dangerous practices occur.

### AN INCOMPETENT CARETAKER.

The nursing question is again to the fore at Cheltenham Infirmary, attention being called at a recent weekly meeting of the Guardians to the case of the unfortunate pauper, an old man of eighty, who, as reported by the local press, met with an accident during the night, shortly before his death. It is not possible for us to decide whether the disaster could have been prevented by the presence of a nurse in the ward, but we venture to say, however that may be, nothing can justify the retention of a half-witted pauper assistant as night attendant on the helpless and sick in an infirmary ward. The inadequate number of nurses has been pointed out more than once to the Cheltenham authorities, and the sooner the deficiency is remedied the better for the interests of the inmates and all who are responsible for their welfare.

### NURSES AND SMALL-POX.

A contemporary, apparently bent anti-vaccination-wards, remarks that "it is often urged that the immunity which is alleged of the nurses in small-pox hospitals is due, and can only be due, to their vaccination," and then deduces from the third report of the Lords' Committee on the Metropolitan Hospitals the fact that the mortality amongst nurses in fever hospitals is very small, drawing thence the wise conclusion that because nurses do not often die of the fevers to which they are exposed, therefore vaccinated or unvaccinated they would not die of small-pox. May we suggest that although at a particular fever hospital not one nurse "died" in ten years, there is no evidence to say that not one suffered from any infectious fever during that time; as a matter of fact, it is by no means uncommon for nurses in general as well as in fever hospitals to contract scarlet and other fevers, though happily deaths are rare.

### NEWLYN NURSING ASSOCIATION.

That the fisher folk of Newlyn appreciate the services of their district nurse is evidenced by the fact reported at the recent annual meeting that they contributed £9 5s. 6d. of the sum of £10 15s. 6d. collected last year towards the expenses of the Nursing Association by Mr. Forster, of the firm of Messrs. Richards, fish buyers. Nurse Goddard, during this her fourth year of work in Newlyn, made over 3,130 visits. This is a heavy record, and we are glad to see that the committee have unanimously accorded Nurse Goddard the addition of £5 per annum to her salary.

### A NURSING HOME AT LEYTON.

The Countess of Warwick, who is one of the vice-presidents of the newly-formed Essex Cottage Nursing Associations, has undertaken to defray the cost of furnishing a nurses' home, which the association intends to establish at Leyton, and to pay the rent annually. These centre homes are of immense advantage where district nursing is concerned, as they insure, if only occasionally, a certain amount of companionship and comfort to workers, who frequently have far too little time to secure either the one or the other for themselves.

### HOSPITAL SATURDAY AT DUDLEY.

The nurses from the Guest Hospital, Dudley, took an active part in the annual Saturday Fund collections the other day. Two stalls and a hospital cot, the latter containing a doll, as representative of the small patients treated at the hospital, ornamented the High Street. The money received by the nurse with the cot was to be specially devoted to the children's ward. Other kind friends of the institution made house-to-house visits in certain districts, and from all the combined efforts made to secure contributions, a substantial result may be hoped for.

### COTTAGE NURSES.

Lady Galway inaugurated a movement recently at Harwath to supply nurses for neighbouring villages. Her Ladyship spoke several times in the course of her speech of the "trained" nurses provided by the Association, and therefore we can indeed congratulate the villages to be benefited. The extreme importance of employing thoroughly trained nurses in cottage homes, where so much is left of necessity to the experience and judgment of the nurse cannot be too often insisted upon. With the low fees asked for the nurse's services, the movement which Lady Galway advocated last week, cannot prove self-supporting, but many generous dwellers in the neighbourhood have come forward to help the Association.

### WORKING WITH THE NURSE.

Connected with the Cottage Hospital at Seacombe is the District Nursing Association. The Associations are fortunate in possessing the services of an excellent and indefatigable nurse. Her arduous duties are lightened in a measure by the kind assistance of ladies in the neighbourhood, who visit with the nurse in the homes of the poor, and give her what aid they can. Such a system could only work well under the best of circumstances, and proves that much kindness and tact is exercised by all concerned.

### DISTRICT NURSING IN ABERDEEN.

Funds are much needed to carry on the work of the Aberdeen District Nursing Association. A system of collecting for this purpose is to be set on foot, from which a helpful result is hoped. The association should meet with plentiful support from all classes in Aberdeen. The nurses have had busy time lately, many serious cases having entailed even more calls than usual on their time.

### NURSING AND KINDLINESS IN BELFAST.

The annals of the Society for Providing Nurses for the Sick Poor in Belfast is delightful reading. The society has indeed lost its esteemed and beloved founder, Miss Macpherson, who resigned control of the institution last autumn. She placed the superintendence in the hands of a small committee. Immediately friends united to secure that her memory should be perpetuated in connection with the society. A large sum was subscribed, and a new depot opened, with nine nurses to carry on the work. The work of the nurses is supplemented in innumerable ways by sympathising friends in Belfast. One lady provides a bed in a cottage hospital for convalescents, whilst another lady sent during last summer convalescent patients for Saturday afternoons into the country, providing lunch and railway fare. Lady Londonderry undertakes a basket for Saturdays in June, and other ladies have promised for other months. The needlework guild provided 2,000 garments for the sick last year. Ladies unite to help to secure three or four weeks' holiday in the country for the nurses. In Belfast the disease most general appears to be consumption, which wields terrible sway in Ireland generally. The nurses are able not only to alleviate suffering in this direction; they can also instruct the poor how to avoid contagion and dissemination of disease.

### NURSES' SITTING-ROOMS.

The recreation or sitting-rooms provided at most hospitals nowadays for the use of the nurses show as plainly as any part of the establishment the improved status of women workers. Something beyond chairs and tables and bare walls is acknowledged as necessary to constitute a suitable "rest" room for tired people. Eyes and thoughts need refreshment as well as weary limbs. Plenty of books and flowers, and a few really good pictures are, generally speaking, quite as requisite as lounging chairs and light couches. And, we are glad to say, we now see all these and many other things provided in the best nursing schools. By way of contrast we read in the *British Medical Journal* Mr. Ernest Hart's impressions of the nurses' quarters at the Station Hospital, Gibraltar: "There is one small day-room for nurses; there is no room attached to each ward, and no little kitchen for comforts; there is no hot water laid on. . . . The four nurses have a very little room for themselves. They have a good deal of running about, as their work is scattered all over the place. The day-room for the nurses has the floor covered with oil-cloth, bare rafters showing, and tempered red walls, and all the fixtures of the roughest kind. The paint in the hospital is dirty, the woodwork rotten, and the whole place bears an aspect of bareness and neglect such as would not be tolerated in any London workhouse."

### SHORT ITEMS.

A man died from exposure to cold in the streets of Madrid on May 23rd, and yet the recent constant changes of temperature in that picturesque capital do not appear to have interferred with vegetation, for we hear of plentiful supplies of apricots, and of asparagus so cheap that for twopence a whole family can have as much as they possibly can consume.—Hinckley is suffering from an epidemic of small-pox, and a galvanised iron isolation hospital has been erected outside the town. Diphtheria, with which the town has been heavily visited of late, is not yet stamped out.—An excellent demonstration was given to the Penrith Women's Sick Nursing Association the other day, on the making of all kinds of poultices and fomentations. At the end of the lecture those present were invited to make each variety for themselves. Accordingly, a busy party was soon to be seen engaged in the various details of their manufacture, whilst Nurse Rudd and Miss Jennings explained their special value in different diseases.—The sum of £82 10s. has been sent by Mrs. Cyril Maude (Miss Winifred Emery) to the Secretary of the Grosvenor Hospital, Vincent Square, Westminster, and the same sum to the Home for the Aged Poor, St. Peter's, Eaton Square, being the proceeds (after payment of all expenses) of the entertainment given by her and other artistes at the Chelsea Town Hall on the 21st ultimo.

### On General Nursing.

By Rowland Humphreys, M.R.C.S., L.R.C.P.Lond.

### XIV.—RHEUMATISM.

Rheumatism is one of those known by the name "General Diseases." It is not confined especially to one part or another of the body, but its effects are spread more or less all over the system. To the eye of a lay person, rheumatism means muscular rheumatism; to the nurse's eye, it means rheumatic fever. Now both of these views are correct, except in that they are limited; whereas rheumatism might be almost said to be polymorphic, in so many forms does it show itself. The two mentioned are the extreme ends of a chain whose intermediate links are formed by such other manifestations of the disease as acute tonsillitis (or quinsy), acute pharyngitis, erythema, and chorea.

The principal characteristic of rheumatism, in whatever form it appears, is that the fibrous structures of the part affected are the ones which principally suffer. Thus, when the joints are inflamed in the course of a rheumatic attack, it is the ligaments which are primarily and mainly touched; the joints themselves are only affected by the disease spreading to the synovial membrane. When the heart is affected the fibrous parts of the valves are the first to be attacked, and from them the inflammation spreads to the heart's lining or endocardium, and then to the heart's muscle.

Rheumatic fever will be described as the type of rheumatism, in which any of the other manifestations of that disease may appear as complications of the more severe complaint, and the other forms of the disease will be mentioned as complications of it.

Rheumatic fever is that development of the rheumatic poison which is betokened by acute inflammation of the joints. It is caused by exposure to wet and cold, and may occur in the course of several specific complaints. It is divided into two forms, the acute or severe, and the subacute or mild.

The acute variety is by far the more serious, for whereas in the mild form no cardiac complication occurs in the greater number of cases, in severe attacks the greater number of cases are left with a more or less severe lesion of the heart's valves.

There are generally symptoms of more or less severity which precede the advent of the complaint, by a period of time varying from three days to three weeks; these are of the nature of sore throat, muscular pains, or erythematous inflammation of the skin. The sore throat is often an attack of acute tonsillitis, and the erythematous attack is usually like one of urticaria, where the swellings on the skin are red instead of white, and are of large size. Sometimes all three of these symptoms are present. Thus, a few years ago, a case occurred in the author's practice, where a sharp attack of papular erythema was followed by one of acute tonsillitis, and this, in its turn, by acute rheumatism, complicated by endo carditis. The sister to this patient was said to be suffering from the same series of disorders at the same time.

An attack of acute rheumatism begins with sharp pain in one of the joints, not uncommonly in the back, the temperature runs rapidly up to 104 deg. Fahr., or thereabouts, profuse perspirations make their appearance, the tongue becomes foul, the bowels are confined, the urine concentrated, and loaded with urates which become deposited on cooling. The pain soon spreads to the medium-sized joints, if they are not already affected, especially those of the lower extremities, and usually the corresponding joints on both sides of the body are attacked at the same time.

The joints are distended by the fluid within them, and there is often a flush on the skin over the more superficial ones. The inflammation spreads from joint to joint, and in turn, in the course of a single attack, all the larger joints of the body and many of the smaller ones, even the spinal joints, may be affected.

The patient lies in bed as if he were helplessly bound by cords, while his mind is usually clear. At times the brain is affected, the mind wandering, becoming childish, or the patient may become maniacal. Curiously enough these symptoms are often associated with inflammation about the heart, as will be further noted later on.

Sometimes the fever runs up to a great height, endangering the patient's life by its severity. It may rise even to 108 deg. Fahr. or more, and at the same time, or just previous to it, the joint pains and the perspirations cease. The patient becomes at first restless, later on cyanosed and comatose, and if some efficient treatment is not instituted the patient will die very rapidly. It is one of those complications which cannot often be foreseen, and in which the nurse must take steps at once to reduce the fever. This is best done by putting the patient in a cold or tepid bath, or by sponging him. Wet packing acts too slowly to be of much use. Salicylate of soda appears to be useless in these cases; quinine seems to act better; antifebrin and antipyrin are contra-indicated. If the temperature be rapidly reduced the patient often recovers, but if allowed to go on unrelieved for any length of time the injury done, especially to the heart, is so great that a relapse is very likely to occur even if the fever be successfully reduced, and then death is likely to ensue.

### Duty versus Danger.

The difficulties in the way of those unfortunate individuals who are charged with the task of erecting fever hospitals do not decrease. The public, not content with throwing every obstacle which objection to site, expense, and non-requirement can present, now seem to demand a practical acquaintance with the details of ward life, when fever is flourishing within the hospitals. A writer to the papers anent the Twickenham Isolation Hospital, thinks the members of the Local Government Board should examine the hospital for themselves, and offers any one who will, the "Annals of Sairey Gamp," as contained in the pages of "Martin Chuzzlewit." There is no doubt whatever that fever hospitals need as efficient supervision as every other kind of institution in which the sick are located, but there should be duly qualified paid inspectors of both sexes appointed for this important work.

### Nursing in New South Wales.

### THE PRINCE ALFRED HOSPITAL TRAINING SCHOOL.

The Prince Alfred Hospital, Sydney, is in a most flourishing state as regards the nursing department. The nurses are thoroughly devoted to their work, and the discipline and general management of the new home are evidences of an ideal training school. At the annual examination in December the nurses all did remarkably well. During their three years of training they have to pass nine examinations, viz., three in each year, and no certificates are given until all have been passed. Prizes of books and small cases of instruments are given to the two best in each grade. The thoroughly excellent training, both theoretical and practical, which is given to the probationers at the Prince Alfred Hospital reflect the highest credit on the Matron, Miss McGahey, and also on the sisters who so efficiently assist her.

### The Mechanism of Movement.

### I.—THE COMPOSITION OF BONE.

The composition of bone is animal and mineral, in very unequal proportions, viz., in 100 parts we have 67 mineral to only 33 animal. The mineral part is the most important, being double that of the animal. There is also an almost inappreciable proportion of fat, viz , one part in 100.

If a bone be boiled sufficiently long, gelatine is obtained, this being the animal matter of the bone. It is also consumed when bone is burnt, and the mineral matter only is left. If a bone be placed in acid the mineral matter is dissolved out and the animal matter alone left.

In a bone the animal and mineral matter are so closely and intimately blended that no amount of care could separate them, and this can only be accomplished by heat or acid. As a proof how closely the two substances are intermingled, whether the bone be treated by heat or acid, it never loses its original form. After burning, what remains is extremely brittle, and must be handled carefully or it will crumble away. After the action of acid, what remains is like gristle, and so flexible that a knot can be easily tied in it.

To this combination of animal and mineral matter in bone is due its great strength. The proportions vary according to age. A young child's bones have more animal matter, and are softer and more flexible than an adult's; hence, if allowed to walk too soon, the weak legs give and become bow-legged. In old people the mineral matter predominates, and the bones are brittle and easily broken. Taking a false step coming downstairs, being pushed unexpectedly off the pavement into the street, turning over quickly in bed, or in cases of great pain clutching hold of the bed or anything else that is at hand will break them. The most important mineral component of bone is phosphate of lime, which alone forms very nearly three-fifths of the whole. It gives toughness to the bone. Shell is made of carbonate of lime, and this with entire absence of animal matter makes it extremely brittle. Phosphate of lime occurs in many foods taken commonly in diet.

It is curious that the bones of our body should vary in hardness. The "long" bones in the extremities are harder that those of the trunk, and the upper bones in both the legs and arms are respectively stronger than those forming the lower parts. The spinal bones, ribs, and the flat bones forming the shoulder blades and collar bones are all about the same hardness, but those forming the skull are the hardest of all. The difference in hardness of various bones is not due to a larger quantity of phosphate of lime, the actual proportions being nearly the same in all bones, but to the way in which the material is put together. Thus "compact" bones are more closely wrought together, and more close-grained than the "cancellous," with the result that we get a structure admirably adapted for the purposes required.

The proportion of mineral matter is, on the whole, greatest when the bones are subject to the greatest mechanical stress, as, for instance, in the shafts of the long bones of the extremities when the bones are also very dense. The strength and density of bones vary a great deal in different persons, and are generally proportioned with great nicety to the weight of the body and to the strength of the person's muscles. In early life the bones are both relatively, as well as actually, small, light, and flexible, and well suited to the nimble and incessant movements of the child, and consequently he escapes many accidents to which his very activity exposes him.

In an adult, when muscular force is greatest, the bones attain their greatest size, density, and strength, and are hardest in those who are strongest and most active.

In old age when muscular and other powers begin to fail, and the movements are slow and cautious, the bones become lighter, more porous, and more fragile.

The balance between the resisting power of the bones and the force of the muscles is the reason why spontaneous fractures are very rare, though they do occur sometimes under unusual exertion, especially with old people. Some people, again, though not old, inherit a very brittle or fragile form of bone and break their bones very easily, the contracting of a muscle or any other trifling cause will effect a fracture with them, but as a rule this occurs with ricketty persons. There are some important points to note with regard to the more minute shapes of bone. The general principle governing the shape of bones is, broadly, that in every case its special form is adapted to the position it occupies and the purpose it has to serve. The middle part or "shaft" of "long" bones is small in circumference to make room for the thick, fleshy bodies of the muscles which occupy the interspaces between the joints. The ends are expanded by a large deposit of this cancellous substance, to make the joints more secure, and to give better leverage for the muscles, and a wider space for their attachment, in addition to the purposes already enumerated. In the shafts, the close packing of the bony material and the support given by the close contact of its particles nearly makes up for the small space upon which the weight is borne, and for the greater disadvantage of leverage against which they must contend. The shaft is strengthened by the ridges and "processes," occurring at the parts where they are most needed, viz., along the hollows of the curves, and here the wall of the shaft is actually thicker.

The bones are rarely, if ever, found to be straight, and the variety of curves and twists in them prevents a stiff motion like wooden dolls, and helps to give our movements ease and our carriage grace. They also give great elasticity to the bones and largely prevent jars being communicated from one part to another, lessening the chances of fractures and dislocations, and of concussion and tearing of the soft parts. The hollow curves are always found on the side where is the largest muscle, which can thus be accommodated in it, and this arrangement gives the smooth, symmetrical outer appearance of our limbs, whilst, were it not for those hollows in the bones, the large muscles would stand out as lumps or ridges on our limbs. The curves are generally greater in short than in tall people, and the greater muscular strength of the former may be considered as some compensation for their smaller stature.

### Appointments.

[It is requested that successful candidates will send a copy of their applications and testimonials, with date of election, to The Editor, The Lodge, Porchester Square, W ]

Croydon Borough Hospital.—Mrs. Maud Evans has been appointed matron of the Croydon Borough Hospital. She was trained at Guy's Hospital, and was matron of Stockton-on-Tees Fever Hospital. We wish her every success in her new work.

East Cornwall Hospital, Bodmin.—Miss Beatrice A. Sharp, who was trained at the Royal Infirmary, Hull, has been appointed matron at the East Cornwall Hospital. Miss Sharp has recently held the post of night sister at the Taunton and Somerset Hospital, and takes many good wishes with her to her new work, in which we cordially unite.

Lewisham Union Infirmary.—Miss Eleanor Letitia Tennent Patteson has been appointed Matron of this infirmary. She was trained in the Nightingale School, St. Thomas's Hospital; was Head Nurse and Night Superintendent at St. Marylebone Infirmary; Night Superintendent at the Royal Infirmary, Bristol, and Surgical Sister at the General Hospital, Cheltenham. Miss Patteson was engaged in private nursing from July, 1891, to July, 1893, and since that time has held the post of Assistant Matron at the Mill Road Infirmary, Liverpool. We heartily congratulate the Lewisham Guardians on having secured the services of a thoroughly-trained lady of such wide and varied experience, and we wish Miss Patteson every success in her new and responsible duties.

### On the Nursing of Enteric Fever in Children.

By the Matron of a Children's Hospital.

Enteric fever is so insidious in its onset, and it is a disease which gives rise to so many complications requiring, especially in the more severe cases, all a nurse's skill and patience, that I purpose in this paper to draw attention to one or two most important points in nursing such cases. They are points with which we, as nurses, are most familiar, because they form part of the everyday routine of our hospital work. But when we are nursing a private patient we may overlook the necessity for carrying out this routine. We should, I think, always bear in mind our reasons for what we do, and I think private nurses would inspire more confidence and would get on much better with the friends of the patients if they displayed more tact, and if they would explain their reasons for carrying out their work in its details.

The bed for a typhoid patient should be a hair mattress covered with bed mackintosh, and under the drawsheet should be another mackintosh. We have been taught that most of the danger of contagion of typhoid arises from the evacuations, so we cannot be too careful to protect the bed on which the patient will have to remain for many weeks. The bed thus protected will enable us more easily and more thoroughly to attend to and keep the little patient comfortable. The patient must be kept lying down, and gently rolled over from side to side for the purpose of changing the drawsheet, and of being washed. He can on no account be allowed to sit up, for we must avoid any sudden strain on the muscles of the abdomen, especially in an advanced stage of the disease.

All soiled linen must at once be removed and placed in a disinfectant (carbolic lotion 1-40). The nurse should carefully inspect each stool and the least trace of blood or any sign of undigested milk should be reported to the doctor. If it should be necessary to save the stool for the doctor's inspection, the bed-pan should be covered with a china cover, and a well carbolised cloth placed over all. No disinfectant should be put into the bed-pan before its use, as this would be liable to change the aspect of the stool, which, before being thrown away, should be disinfected with either carbolic powder or carbolic lotion—1-20.

We know that the poison of typhoid attacks the intestines, the mucous membrane and the mesenteric glands becoming inflamed and the intestines ulcerated, hence the necessity for the great care in giving nourishment, and for keeping the patient absolutely quiet. We must always bear in mind that the ulcers take a long time to heal, and until this healing has taken place we are to fear hæmorrhage and perforation.

I wish to lay great stress on the necessity for the nurse to wash her hands thoroughly after attending to a typhoid patient. A weak solution of Condy's fluid is a good disinfectant for this purpose, but plenty of soap and water is sufficient. I think a nurse cannot be too particular in this matter; it is due to herself that she should be most careful that by no fault on her part she should contract the disease from her patient.

As this fever is a very exhausting one the patient often becomes greatly emaciated, in which case a water bed will be found most comfortable. Attention must, throughout the illness, be paid to keeping the back and all bony prominences dry, and to preventing bed sores. Where there is redness a ring pad will be found useful to prevent any pressure on the bony prominences. The lower part of the back and the buttocks may be rubbed with zinc or boracic ointment.

The room should be well ventilated, and the covering light but warm. The patient should be carefully sponged all over night and morning with tepid water, and general restlessness is often overcome by frequently sponging the face and hands. This illness is such a tedious one, especially in the convalescent stage, that we should do all we can to relieve the weariness and make the patient as comfortable as possible, at the same time preventing any excitement.

With regard to the diet. This and the quantity will be regulated by the physician, the nurse's duty on this point being to see that the nourishment is assimilated. Should there be flatulence or undigested milk (curds) in the motions the doctor's attention should be called to the fact.

The temperature, which is important, should be taken every four hours, or oftener. With children the temperature should always be taken in the rectum, the thermometer being put into vaseline before inserting, and afterwards kept in a carbolic solution—1-20. The pulse and respiration should also be taken every four hours, for by these means we are able to note the beginning of any complication; for instance, if we get a sudden fall of temperature we fear hæmorrhage, and we may get this fall of temperature before there is any evidence of hæmorrhage in the motions. Also, if we have an increase in the respiration we may suspect pneumonia, a not uncommon complication. I mention this to show the importance we attach to the temperature, pulse, and respiration in this disease. By these means, and without causing any annoyance to the patient, we are often able to recognise the onset of these complications, and we are thus able to report to the physician.

In this disease we get either constipation or diarrhœa. In the former case, should an enema be ordered, it must be given most carefully.

There are many complications—hæmorrhage, perforation, peritonitis, pneumonia, &c. We must also be very careful that we do not get a relapse, for in many cases this is more serious than the former part of the illness. A severe case of typhoid fever is a great strain on the nurses, and they owe it to the patient as well as to themselves that they should get as much fresh air as possible, and so keep themselves in health. They should always have a good meal before going on duty, and never eat anything in the patient's room. The mattress and blankets should be stoved after the illness is over, but the doctor's wishes must be ascertained with regard to this and any other means of disinfection.

### Notes and Queries.

### Queries.

(70) *Disinfection.*—Will you kindly give me information as to the best method of purifying bedding used in infectious wards?—*Enquirer.*

(71) *Chorea.*—Can you tell me of a hospital or convalescent home by the sea where a little girl suffering from chorea would be taken?—*District Nurse.*

(72) *Consumption.*—Is there any Home of Rest for gentlewomen with this disease in England?—*F. G.*

(73) *Song.*—Will you kindly tell me where I can find the words of a song beginning "Though poor be the chamber"?—*Sister Kathleen.*

(74) *Nursing Manual.*—I shall be glad to know what book you recommend.—*Nurse Mary.*

(75) *Training.*—A Head Nurse of some years' experience in an institution wants to know whether she can undertake to train probationers, as she is not herself a trained nurse.—*Head Nurse.*

### Answers.

(70) *Disinfection* (*Enquirer*).—Read "Sanitation for Nurses," especially chapter xiv., which came out in The Hospital of July 22nd, 1893.

(71) *Chorea.*—You had better write to the matron or secretary of some of the homes given in "Burdett's Hospital Annual."

(72) *Consumption* (*F. G.*)—A little Home of Rest has been established at St. Kenelm's, Great Malvern, where four ladies are received at a time, lodged free of charge, and board themselves. Cases of phthisis in the early stage are admitted. We believe the home was established by a lady who, having recovered from consumption in the Malvern air, desired that others, who perhaps could not afford to take lodgings, should benefit as she has done.

(73) *Song* (*Sister Kathleen*).—The words occur in Gounod's "Nazareth."

(74) *Nursing Manual* (*Nurse Mary*).—You had better write to the Scientific Press, 428, Strand, and get them to send you a catalogue of their nursing books.

(75) *Training* (*Head Nurse*).—No untrained person can possibly undertake to properly train probationers, although no doubt the head nurse who asks this question is fully competent to teach many useful things.

### English Nurses in Brazil.

(*By a Correspondent.*)

RIO DE JANIERO.

I.—The Strangers' Hospital.

According to a long-standing promise, I send news of the hospital and work here to the numerous readers of this journal. So much has been written, and so many illustrations given during the late revolution, that when I tell you the Strangers' Hospital is situated on the spur of one of the hills lying between the Sugar Loaf and Corcavado, you will see we live in one of the most beautiful spots of this lovely harbour. To the right the grand old Sugar Loaf, to the left the chain of mountains commencing with the Corcavado, whilst in front the harbour forms a wonderful panorama, with the Organ Mountains standing out in bold relief. Looking upon these glories of nature, it seems hard to believe that so much suffering exists in the midst of it, yet year by year the yellow fever fiend stalks through and claims victims by the thousand.

For many years the want of a hospital for English and American residents was seriously felt, numbers of young men of both nations being employed in the banks and business houses. Encouraged by a very handsome subscription from the London and Brazilian Bank, a scheme was put in shape, and in a very short time £15,000 sterling was raised by the English and American houses and banks.

A suitable site at a convenient distance from the city was sought, but nothing available for building land could be found. A large residence standing in its own grounds was eventually offered and gladly taken by the committee, and by the aid of a skilful engineer (Mr. Randall Callender) this old residence was transformed into a model hospital. Two very handsome towers were added, improving the appearance, and containing bath-rooms and lavatories, all fitted up with the latest improvements for the supply of hot and cold water.

A new wing was added to the west side of the building increasing it to accommodate fifty patients at present.

The largest ward runs the full length of the hospital and contains ten beds; the other wards hold from four to eight. There are also several private wards.

It was originally intended to nurse medical cases only on the ground floor, and to isolate yellow fever in the upper part. The building was therefore constructed without any communication between these two floors except by speaking tubes. This answered very well the first season, but during the last season's epidemic the whole building was utilised for cases of yellow fever, reserving one room as an observation ward, where doubtful cases were placed for 24 hours. New kitchens and servants' offices were built, making the whole a most comfortable and imposing place. The staircases, polished landing, and hall are very much admired, and so is the great cleanliness prevailing.

Great disappointment and annoyance were caused to the committee when, after all was ready, they were forbidden to open the building as a hospital because of its close proximity to the military school. The objection was raised by the health authorities. Application was made to the President (Floriano Peixoto) and his inspection invited. He most kindly responded and visited the hospital, and not only approved but admired the whole thing, and granted permission to open it, a matron and the sisters being already on the spot. The ceremony took place on Sunday, January 8th, 1893, it being convenient for business people to attend the ceremony on that date. It was quite an imposing sight, quantities of flowers, ferns, palms, &c., were sent in the day before. Every place was well decorated, and a large company of English, Americans, and many Brazilians assembled, and some eminent medical men, including Dr. Bento, the great authority on yellow fever. The Rev. Henry Mosely made the opening speech, and several followed, both in Portuguese and English. The President's health was drunk with great zeal, and then the engineer responded to a speech made on behalf of the ladies who had come out to do the nursing. Visitors rambled all over the place, and after partaking of light refreshments they departed.

For the first few months we occupied rooms in the hospital until the *châlet* for the nursing staff was finished. This is a very nice six-roomed house, with bath-room, &c., all very nicely fitted. Each nurse has a separate room, and there is a sitting-room and verandah commanding a lovely view. The matron's quarters are in the main building. The grounds are very extensive, and very prettily laid out like a private park, and there is a laundry and a mortuary. All the wards are nicely furnished, reminding visitors more of a private house than a hospital. There are lockers, also large baths on wheels for fever cases. The bedsteads came out from England, and instead of counterpanes they are covered with white blankets, with "Strangers' Hospital" woven in blue letters. The effect is pretty to visitors, but upon patients the words have rather an irritating effect. They will try and form other words with the letters, especially when the clothes get tossed about and the letters seem to group into different words. These blankets, with all the linen ready made for thirty beds, were the handsome gift of Messrs. Edward Ashworth and Co.

Everyone assisted the hospital in a most liberal and energetic manner. The English ladies got up two bazaars which realised several hundred pounds; then a grand ball was given, which raised over two thousand pounds; many wealthy Brazilians took part in it, and showed the most friendly interest in it all. I went to this ball in uniform—grey print dress, cap, and apron—and many persons had never seen a nurse in uniform before. This ball was held in Petropolis, the great diplomatic centre of the foreign portion of the community, that is to say, English, Americans, French, Germans, &c. In the days of the Empire the Emperor lived there. The palace still remains.

### Nursing in Switzerland.

We have been asked to give an opinion as to the prospects of English nurses going out to Switzerland "on their own account," and we are glad to be able to put before our readers the words of a correspondent who is particularly well qualified to speak on the matter: "Private nursing in Switzerland is not likely to prove lucrative, for the expenses of travelling and residence are very heavy. Then the demand for nurses is very variable. There are few English practitioners, and the Swiss doctors, in most cases, naturally choose Swiss nurses. There is also a difficulty in bringing English nurses and patients together, yet it sometimes happens that the presence of a good English nurse would be invaluable. Certainly no nurse should go to Switzerland unless she can afford to pay her own expenses. She might eventually do well, especially at Zermatt or Portresina. The summer season in the mountains lasts from about the middle of June to the middle of September."

### Certificated Asylum Attendants.

BERRY WOOD ASYLUM, NORTHAMPTON.

Nursing Certificates.—The final examinations at the close of the winter lectures and instructions (conducted by Dr. Harding) took place recently, when the following nurses obtained the certificate: Nurse Margaret Spencer (silver medalist), Nurse Henrietta Senside, Nurse Elizabeth Rylett, Nurse Minnie Bateman, and Nurse Catherine Edwards. To gain these certificates three years' continuous residence at Berry Wood and attendance on lectures and instruction are necessary. Each examination consists of one day's written, one day's oral, and one day's practical examination. The certificates are signed by the examining medical officers and by three of the committee of visitors.

### District Nurses.

By A Dispensary Physician.

The argument of a " Country Practitioner " with regard to the question of practical finance in obtaining hospital-trained district nurses is very convincing, but not altogether consistent. In the first place he enlists our sympathies for the impecunious parson and squire in being required to provide the sum of £70 or £80 per annum for a properly-trained district nurse, but in the latter part of his letter we find these much-to-be-pitied individuals are not to find the money for the cottage nurse, but she is to become an official under the control of the county district or parish council, and herein lies a danger not only to nursing as a profession, but to the community at large.

On the principle that a cheap and inferior article is better than nothing at all, it is proposed that the County Councils shall support, and officially recognise, as the nurses of the sick poor, a body of women who, in the words of the honorary secretary of the Dorset Health Association, " have received a course of instruction in the principles of nursing, but not such as to fit them to be considered adepts in any branch of the profession." That is to say, a low standard is to be adopted, and our poor, who really require more skilled nursing than the well-to-do, are to take what they can get and be thankful.

It is urged that these women will not be looked upon as "professional" nurses; well that may be so, but depend upon it, whatever her official title and training may be, the woman who wears a nurse's uniform, and works in a district, will be spoken of and recognised as " the nurse " by those among whom she labours, and in a very short time will come to consider herself as such. I have had fifteen years' experience among properly trained and imperfectly trained nurses, both in hospital and in district work, and have almost invariably found the imperfectly trained nurse more self-assertive and more likely to act injudiciously in the interests of her patients, than her fully-trained sister, who knows what " intelligent obedience " really means.

" A Country Practitioner " writes in such a satirical manner of the requirements of a hospital-trained district nurse, that I can only think his knowledge has not been obtained from personal observation.

I have tried both fully-trained and three months'-trained nurses. Three years ago I worked with the latter, but gave them up after a fair trial, and for some time worked alone, preferring to do without nursing aid at all, rather than be responsible for the work of women in whom, from their insufficient knowledge, I could place no reliance. Since then I worked with hospital-trained district nurses, and it may be interesting to your correspondent to hear that we only send acute cases into hospital when the strain upon the resources of the family would be too great, or the friends are too ignorant to be trusted with the carrying out of directions between the visits of the nurse.

I find the nurses take the keenest interest in their work, and do not attempt to pose as medical students, and if they do perform menial duties, it is in a quiet and unostentatious manner.

It is not just to hospital-trained district nurses to assert that they care only for " interesting " cases. My experience has shown me they are just as careful and painstaking over such trivialities as ulcerated legs, measles, influenza, &c., as when nursing typhoid fever or pneumonia. Measles, by-the-bye, is far from being classed as a trivial complaint among the poor, as the death-rate clearly shows, and needs very close attention to avoid the occurrence of unpleasant sequela.

I once heard a nurse remark, when going round with the regular nurse, that she did not see the necessity of " walking a quarter of a mile simply to rub a bronchitis case with liniment." This same nurse had been trained in a workhouse infirmary, and probably belonged to the class from among whom the cottage nurses will be drawn. Further comment is, I think, needless.

Even if, as your correspondent states, country practitioners as a rule send their acute cases into the county or cottage hospitals, many cases must arise where the patient cannot be moved from home. In such a contingency will the parson and squire, or even the parish council, agree to pay two guineas a week for the services of a fully trained nurse, when the cottage nurse is available? I trow not, and the result will be, that the unfortunate woman will be compelled to undertake a responsibility for which in all probability she is both mentally and technically unfitted.

This sense of responsibility, which simply means the power of intelligently carrying out the doctor's orders, has been so felt by some of the district nurses, who unwisely were content with only one year in hospital, that they have decided to return to hospital work for another year, in order to fit themselves more thoroughly for their work.

One great advantage in training educated women for district work is that they are able more efficiently to act as teachers, more especially as regards questions of sanitation.

Women of the class it is proposed to institute as cottage nurses, will probably find much difficulty in imbibing even the elementary principles, and are not likely to be able to impart them intelligently to others.

It may be urged that the teaching of sanitation, &c., should be through the lecturers of the health associations. I am a member and a supporter of the National Health Association, but I know this, that in spite of the advantages of the present Education Act, it is exceedingly difficult to impart information to people who are incapable of retaining more than one fixed idea in their heads at a time. In this I speak feelingly, for I have wasted many hours in the attempt.

Mr. Burdett, in his enthusiasm, dilated too much upon the necessity of a donkey or a tricycle as a means of locomotion for the trained district nurse; and the nurses are by no means the weak-legged creatures a " Country Practitioner " would have us believe, indeed, I know of nurses now, who are walking quite as far on the hard unyielding pavements in the suburbs of London as they would have to do in any country districts.

The question of ministering to the wants of our sick poor is a national one, and if a scheme of nursing is to be one of the features in the programme of our parish councils, I earnestly trust that rather than start with a low standard of nursing, thereby flooding the country with women but ill equipped for the very important work they are called upon to perform, calm deliberation will be exercised, and that, at any rate at first, efforts will be made to maintain that high standard to which, after years of patient struggling, nursing as a profession has been raised. We cannot afford to do anything that would tend to a return to the days of the Mrs. Gamps, who have long since ceased from troubling.

As I have stated before, the poor stand in need of nursing even more than the well-to-do, and it is unjust on the face of it to offer to them in their hour of need a form of assistance which we should deem inadequate for ourselves.

### Minor Appointments.

West Ham Hospital, Stratford, London. — Miss Maude Clenel, who was trained at this hospital, was recently appointed Nurse in Charge of the out-patient department at the same institution.

### Practical Suggestions.

### A TEN CENTIME PIECE.

Some years ago, when living in Belgium, I had the pleasure of knowing the wife of the "pasteur" of the French Protestant Church in a small town not many miles from Brussels, and I found that one of the numerous excellent rules which governed the daily routine of her methodical and charitable life was to give a ten centime piece as an honorarium to anyone who brought her a good idea. I remember one day this sum was given to a small, pale-faced girl, who had come, as her weekly treat, to have her Sunday tea at the parsonage after a hard week's work as a dressmaker's apprentice. She had suggested that each member of the small congregation should give at least one garment a year for the poor.

"That is a good idea, Adèle," said Madame approvingly, "and no doubt it can be carried out. We will try to have the garments ready before the cold weather comes."

The congregation was not a rich one, but the claims of the poor were never forgotten, and every Sunday at the close of the service, when the people stood up to receive the parting benediction, the accustomed words fell on their ears, "Souvenez-vous en sortant de vos frères les pauvres," and on coming from the humble building which served as their church they found on the top of the winding stone steps which led down to the steep and narrow road, the "pasteur's" eldest son, who stood with an alms-dish to receive the small sums.

To secure a good idea for ten centimes is an excellent bargain, and one does not often get so much for one's money. We constantly hear of sales, and of great reductions made on all kinds of things, but ideas are not plentiful in any market, and they are worth a fair price.

### Nurse Nora's Good Idea.

A year or two ago, Nurse Nora, having left the hospital at Uptoyne and gone out to nurse on her own account ("to make a little money for her old age"), heard from her friend, who had charge of it, that (in company with many other excellent places) they were much in debt, and would have to be very careful in their expenditure that year. "And," added the sister-in-charge dolefully, "Christmas will soon be here, and we want so many things —our bed jackets are all worn out, and how can we get new ones to make the patients look nice? I could not bear to see the poor things wearing shabby ones on Christmas Day. If Santa Claus would only bring us a great bundle of flannel!"

Nurse Nora sat down with a worried expression.

"Sister must have her jackets," she said. "I can give a little. I might spare five shillings; that would help. But that is not much use by itself. I must think of something better than that." Great ideas were coursing through her brain; they must be pursued and caught, and the useful ones kept in leash for future use. Several were followed up, but found when secured not to be what she was in search of. At least, one was grasped, which seemed nothing short of an inspiration.

"Quite easy," she murmured, "and sure to answer. Sister shall have her jackets, and all the patients shall look as grand as red flannel can make them. An army of red coats. 'Sister's Own' regiment; grand parade." Nurse Nora laughed to herself. "No," she said, with great firmness, "I shall not give the 5s. I shall spend it on something else."

In a few days the something else took form in the shape of a packet of cards neatly printed, with a choice design by Nurse Nora herself. Each card was headed, "Uptoyne Hospital Needlework Guild," and a space was left for the name of the owner of the card, who on becoming a member of the guild undertook to make at least two garments a year for the patients in Uptoyne Hospital. This was asking so little that it would be difficult for anyone who had not a heart as hard as Pharaoh's to refuse. At any rate, no one did refuse Nurse Nora.

Perhaps she had a happy way of asking, and there is a great deal in that; it is quite an art in itself. There are many things to be remembered in making a petition, however trivial, if one wishes to succeed: "Whom to ask, how to ask, where to ask, when to ask"; and all these points Nurse Nora had evidently carefully considered; and as she always took it for granted that it was a pleasure to belong to her useful guild, others seemed to find it so too, and the cards soon found owners. An old lady whom she had nursed quite "took to" the idea, and found several members among her own friends. Her two grandchildren were proud to have their names enrolled as members of the guild, and took cards to their school to "make other girls join."

It is strange how many people seem only waiting to be asked when help for others is wanted. They are quite willing to do their share, but would never have done it by themselves.

"A very good idea says one of these passive personages. I wonder I never thought of it before."

I wonder too, for it does not require any very great mental effort to discover that there are many people to be helped in the world, and much waiting to be done, and so the burden and strain fall on the shoulders of the willing few, while others stand all the day idle, not so much indifferent to the claims of others, as regardless of them until they are specially pointed out to their notice. "Evil is wrought by want of thought as well as want of heart." That was the beginning of the work, and the end of it (not the real end, for the work is still going on) was that on Christmas Day, the Sister-in-charge at Uptoyne was a proud and happy woman, as she looked at the rows of patients in their "brave attire." Her pride and happiness being only equalled by the feelings of Nurse Nora, who never tired of feasting her eyes on the brilliant result of her good idea. It was an excellent idea, and would have been ridiculously cheap at ten centimes, and now The Hospital readers have got it for nothing. M. G. Smith.

### Our Own Good Idea.

For years past one of our own specially good ideas has been freely placed at the service of all Hospital readers. We mean Our Annual Needlework Competition, which has enabled us to supply not only red jackets, but many other useful garments to members of the sick poor who spend Christmas in hospital wards. We hope the number of contributors will go on increasing every season. Each new competitor is one of a circle of relations, workers, and well-wishers, and, therefore, for everyone who joined us in 1893 we may certainly count upon at least half-a-dozen more in 1894. Good ideas being like sound seed will assuredly result in excellent crops of deeds!—Ed. T. H.

### Presentations.

Addenbrooke's Hospital, Cambridge.—On May 27th, the anniversary of her birthday, the probationers of Addenbrooke's Hospital presented Miss Esther Young, the Assistant Matron, with a handsome silver cream jug and sugar tongs, and with an illuminated card expressing their good wishes. This token of their esteem is well merited, for during the two and a half years Miss Young has held the office of Assistant Matron she has been most self-denying and indefatigable in her efforts to promote the comfort and happiness of the nurses in every way. It is entirely owing to her exertions that the ward for sick nurses is now as tasteful and luxurious an apartment as the most fastidious invalid could desire, containing everything necessary for the alleviation of suffering as well as for beguiling the tedium of convalescence.

☞ For The Muses' Looking Glass, Everybody's Opinion, Book World for Women and Nurses, &c., see page lxxxix. et seq.

### The Muses' Looking Glass.

### UNDER THE RED ROBE.*

Mr. Weyman has not kept us waiting long for another story, and we think that "Under the Red Robe" will prove as attractive reading as "A Gentleman of France." It has many points in common with its predecessor; again the hero is a noted duellist and a finished swordsman, and the terror of the gambling places and restaurants which he frequents. At first he is somewhat of a bully and swash-buckler, but gradually the higher side of his character develops. Some of the scenes are masterly, notably the duelling scene, in which Gil de Berault, the hero, fights a young Englishman who has had the hardihood to accuse him of cheating at cards.

Duelling has been prohibited in Paris by Cardinal Richelieu, death being invariably the penalty assigned to the man killing his adversary. In spite of this de Berault insists on fighting, and from the first the Englishman has no chance against the practised Frenchman until (it is de Berault who speaks) "I slipped and was down in a moment on my right side, my elbow striking the pavement so sharply that the arm grew numb to the wrist. He held off. I heard a dozen voices cry, 'Now! Now you have him!' But he held off. He stood back and waited with his heart heaving and his point lowered until I had risen and stood again on my guard. 'Enough! Enough!' a rough voice behind me cried. 'Don't hurt the man after that.'

"'On guard, sir,' I answered, coldly—for he seemed to waver and be in doubt.

"Several voices cried 'Shame!' and one 'You coward!' but the Englishman stepped forward, a fixed look in his blue eyes. He took his place without a word. I read in his drawn, white face that he had made up his mind to the worst, and his courage so won my admiration that I would gladly and thankfully have set one of the lookers-on, any of the lookers-on, in his place; but that could not be. So I thought of Zaton's closed to me, of Pombal's insult, of the sneers and slights I had long kept at the sword's point; and pressing him suddenly, with a heat of affected anger, I thrust strongly over his guard, which had grown feeble, and ran him through the chest.

"When I saw him lying laid out on the stones, with his eyes half shut, and his face glimmering white in the dusk—not that I saw him thus long, for there were a dozen kneeling round him a twinkling—I felt an unwonted pang. It passed, however, in a moment, for I found myself confronted by a ring of angry faces—of men who, keeping at a distance, hissed, and cursed, and threatened me, calling me 'Black Death,' and the like.

"They were mostly canaille who had gathered during the fight, and had viewed all that passed from the farther side of the railings. While some snarled and raged at me like wolves, calling me 'butcher' and 'cut-throat,' or cried out that Berault was at his trade again, others threatened me with the vengeance of the Cardinal, flung the edict in my teeth, and said with glee that the guard were coming, they would see me hang yet.

"But I cocked my hat, and drawing my cloak over my shoulders went out with a swagger, which drove the curs from the gate before I came within a dozen yards of it. The rascals outside fell back as quickly, and in a moment I was in the street."

But at this moment the Cardinal's guards come up and close round him and carry him off. He, however, manages to effect a compromise with the Cardinal by undertaking to bring alive to Paris M. de Cocheforêt, a noted rebel and outlaw. To fulfil this he becomes the friend of the wife and sister of the unfortunate man, and is at first quite untouched by any scruples of honour in the matter, but presently he realises what a despicable part he is playing towards the two noble and trusting women, and he is conscious of a warmer feeling in his heart than mere friendship for Mdlle. de Cocheforêt.

This gives Mr. Weyman scope for some fine character writing and for exhibiting the masterly manner in which he can rescue his hero from an apparently inextricable difficulty, for the Cardinal has ordered a detachment of dragoons to go to assist de Berault in running his prey to earth, and these dragoons are quartered in the castle of their victim. Here they behave abominably to M. de Cocheforêt's wife and sister, and all the latent nobility of de Berault's character is awakened to act in their defence, and thus the two emissaries of the Cardinal are at open variance. The officer of the dragoons thinks to ruin de Berault in Mdlle. de Cocheforêt's eyes by proclaiming him a spy and traitor and so forestalls the confession which de Beraultt had intended making to her. We will let de Berault describe his own feelings: "A score of times I had thought with shrinking how I should reveal my secret to mademoiselle, what I should say, and how she would take it; but in my mind it had been always a voluntary act, it had been always I who unmasked myself and she who listened—alone; and in this voluntariness and this privacy had been something which took from the shame of anticipation. But here was no voluntary act on my part, nothing but shame. And I stood convicted, speechless under her eyes—like the thing I was."

This scene is a fine one, but we must leave the reader to learn for himself how both come out of the ordeal and how the story ends.

The directness and terseness of Mr. Weyman's style are decidedly refreshing, and he is one of the few writers who can successfully use the first person singular throughout his narrative.

* "Under the Red Robe." By Stanley Weyman. (2 Vols. Methuen and Co. 21s.)

### Where to Go.

Stamford.—On Saturday, July 7th, a jumble sale in aid of the funds of the Stamford Branch of the Queen Victoria Jubilee Institute will be held in the Stamford Town Hall. Gifts of useful and serviceable articles will be gladly received for the sale. Address Miss Sewell, hon. secretary, 59, St. Martin's, Stamford.

Princes' Hall, Piccadilly.—On Thursday, June 21st, a grand concert, under the immediate patronage of H.R.H. the Princess Louise, will be given in aid of the National Orthopædic Hospital, Great Portland Street. The following artistes have generously given their services: Madame Clara Samuell, Mdlle. Antoinette Trebelli, Madame Antoinette Sterling, Mr. Ben Davies, Signor Foli, and the Meister Glee Singers.

Queen's Hall, Langham Place.—"Mrs. Brewer's Afternoon," in aid of the North-Eastern Hospital for Children, and other charities, is fixed for Thursday, June 7th, at three p.m. The programme is exceptionally good, and includes songs by Madame Antoinette Sterling, Madame Alice Gomez, Signor Caprille, "The Scandinavian Quartette," The Schartan Part Singers, solos by Mons. Hollman and Mr. Vivian Herbert, and Mr. Brewer will preside at the organ. Recitations will be given by Mrs. Sarah Grand, Mrs. Albert Barker, and Miss Gethen (late Sister Queen). Special tickets for nurses at a shilling each can be obtained by immediate application to Mrs. Brewer, 21, George Street, Hanover Square.

St. Elizabeth's Hospital, Great Ormond Street.—An entertainment, consisting of living pictures and music, will be given by the Dowager Duchess of Newcastle at 15, Hill Street, Berkeley Square, on Tuesday and Wednesday, June 12th and 13th, at ten p.m., in aid of St. Elizabeth's Hospital and other charities. Tickets, price one guinea, may be obtained from the Secretary, Dowager Duchess of Newcastle, 15, Hill Street, or Mitchell's Royal Library, Bond Street, W.

### Everybody's Opinion.

### UNTRAINED BUT ACCEPTABLE.

"A Trained Nurse" writes: The admirable article recently published in The Hospital on "District Nursing" is a valuable addition to the excellent series on the same subject written, I believe, by a superintendent of experience, which appeared earlier in the year in the "Nursing Mirror." Such practical papers are of immense service to nurses, and they also give us more encouragement than the writers perchance realise. Of late we have heard so much of respectable and uneducated women being such desirable attendants for the rural poor that we, who have been at considerable pains and expense to fit ourselves for what we have been taught to consider the highly responsible position of district nurses, have felt a little disheartened. It seems almost as if the contract system would shortly be applied to nursing, and any "tender" accepted, which undertook the supply of the largest number of women at the lowest possible rate per head. What matter if these worthy persons have had but a few weeks' instruction in "the principles of nursing?" They are *cheap*! And the sick poor (alas!) dare not "look at a gift-horse" too critically. However, it is amusing to notice how entirely these mushroom associations are in the hands of pure amateurs, with the occasional assistance of a lady or two, who has had three months' hospital experience, or once gained a St. John's Ambulance certificate, the well trained nurse or experienced superintendent of district nursing being apparently carefully excluded from giving her opinion on this most important subject. The thanks of all qualified district nurses are due to the editor of The Hospital for kindly opening his columns to discussion.

### KIMBERLEY HOSPITAL.

Dr. H. Draper Bishop, late Senior House Surgeon, Kimberley Hospital, writes from Glendalough, Weston-super-Mare: As the Matron of this hospital, Sister Henrietta, is at present in England, and is, I believe, anxious to procure some nurses from England, to add to her staff, it would be well if nurses who have any idea of going to Kimberley understood the system that is in vogue at this hospital before finally deciding to leave this country. The Hospital Board pay a lump sum to the "Community of St. Michael and All Angels" (of which Sister Henrietta is the head at Kimberley) to find nurses for the hospital, and having done this decline to be answerable in any way for their nurses; they equally ignore any complaints made by nurses, or any suggestions for their welfare. In the case of injustice being complained of, there is said to be an appeal to the head-quarters of the order at Bloemfontein, but as far as I know, no notice of any complaint has ever been taken, so that practically every nurse is entirely in the hands of the Matron, and whether this is a good system or not I leave your readers to judge. When 6,000 miles from her home, and perhaps with no friends in the country, it is of the greatest importance that a nurse should know that her interests are protected by a just and impartial board, which unfortunately is not the case at Kimberley. Articles have lately appeared in nearly every paper in the Colony severely criticising the management of the hospital, and it is highly probable, according to the *Cape Times*, that an inquiry into the condition of this and other hospitals in the Colony will be held when Parliament meets again. The dress of the nurses (it cannot be called uniform) consists of a black unwashable stuff dress, made in any fashion, and of any material that the wearer may desire; this is worn both in summer, when the temperature is often 100 F. in the shade, and in the winter, when it is bitterly cold. The hours of duty are long—from seven a.m. to nine p.m. on day, with two hours off, and from nine p.m. to seven a.m. on night. What the salaries paid are I cannot say, only the Sister is in a position to do so; but they should be good, as the Board pay £180 to £190 a month for about forty nurses. The hospital "private staff" has no connection with the hospital; it is managed entirely by the Sister in charge, who herself pays the nurses she employs. I cannot say that the nursing of coloured patients is a disadvantage; to their nurses they show every respect, and their tact and delicacy are remarkable. If their white sisters would only treat those around them with as much consideration, Kimberley Hospital would be a happier place than it is at present. Having just returned from Kimberley, I am in a position to give any of your readers who may desire it, further particulars, and I shall be pleased to do so.

### Roden Noel.

By One Who Knew Him.

On Saturday last, while travelling in Germany, the Hon. Roden Noel died suddenly of heart disease. Well known as a poet and as a litterateur, there was another side to Roden Noel's character only known to a small circle—chiefly included amongst the readers of this journal. For Mr. Noel was a philanthropist as well as a poet; his sensitive nature felt as keenly all the miseries as all the beauties of this world, and many a hospital ward will miss that striking figure—that worn face with the sweet smile—which used to linger specially round the children's cots. The Nurses' Co-operation has lost one of the most valued members of its General Committee, and the District Nurses at Brighton have to mourn one of their chief supporters. The younger son of the first Earl of Gainsborough, Roden Noel received the usual education of his class, and graduated at Cambridge in 1858. But though he held a post at Court, for some years he ever found his chief pleasure in the society of his brother poets, and he loved best the somewhat Bohemian atmosphere of Robert Buchanan's study. In 1880 Roden Noel lost his youngest and best-beloved child, and sorrow stung his fine nature to its finest issues. The following year he published a volume of verse, called "A Little Child's Monument," which far exceeded any of his earlier works; it stands only second to "In Memoriam" for brooding tender sentiment and for true religious feeling under bereavement. One verse can show how perfect is the lyrical faculty, how pathetic the expression of sorrow:—

I am lying in the tomb, love,
 Lying in the tomb,
Tho' I move within the gloom, love,
 Breathe within the gloom!
Men deem life not fled, dear,
 Deem my life not fled,
Tho' I with thee am dead, dear,
 I with thee am dead,
 O my little child!

In contrast to the delicate ring of the above lines was the whole of the socialistic little pamphlet, called "Poor People's Christmas," and brought out in 1890. The rugged versification was there adapted with power to the rough subjects under treatment. The effect was startling—almost unpleasant—but there could be no doubt that the technical workmanship was grand. And so it continued to be, some of Roden Noel's best work appearing in the Canterbury series quite lately. With rare generosity he added to the re-prints therein contained some new sea-lyrics, the scheme and rhythm of which were perfect, and fully showed the love of the sea, which he, as a swimmer and a poet, shared fully with Byron. One of Roden Noel's finest qualities was his appreciation of his fellow-craftsmen and his joy in their work—shown specially in his essays and in the volumes of Spenser and Byron which he edited. Only on May 8th Mr. Noel was in London reading a paper on Sir S. Fergusson's poetry before the Irish Society. Asked then to read some of his poems at Mrs. Brewer's afternoon on June 7th in aid of a children's hospital, he replied with his usual extreme kindness in the affirmative, writing afterwards to say that a journey to Germany would prevent him keeping the appointment, but that if at any other time he could help he would gladly do so. Alas! the journey proved too much for a heart which rheumatic fever had left affected, and Roden Noel passed suddenly away from us, the blow falling with such swiftness there has not yet been time to realise our loss.

### Wants and Workers.

Will someone kindly tell me of a Home where a little boy, aged five years, and subject to epileptic fits, would be received.—*A Friend.*

### The Book World for Women and Nurses.

[We invite Correspondence, Criticism, Enquiries, and Notes on Books likely to interest Women and Nurses. Address, Editor, The Hospital (Nurses' Book World), 428, Strand, W.C.]

Esther Waters. A Novel by George Moore. (London: Walter Scott.)

This story has lately acquired an adventitious notoriety by reason of the somewhat extensive controversy carried on in the columns of the *Daily Chronicle* and other papers regarding the justness or the reverse of its exclusion in the interests of morality from the shelves of Messrs. W. H. Smith's circulating library. By an almost unanimous verdict the book has been declared innocent, and the librarian found guilty of high-handed and unjustifiable censorship. The story is essentially one of low life, and deals with the temptations, struggles, and folly of a young maid servant, Esther Waters, in the service of a country gentleman addicted to the pleasures of the stable and the turf. The example which the master sets above stairs is closely imitated by the servants in the kitchen, and thus in the first third of the story we are introduced to the quarrels and jealousies of kitchen life, the betting and drunkenness of the stables, and the wooing and betrayal of Esther Waters by William, the footman, whose handsome face and broad shoulders have attractions also for one of the young ladies of the household—an infatuation which terminates in Esther's discomfiture and a runaway match between the footman and his young mistress. Esther loses her place, and after many vicissitudes in London, and after many temptations which are, perhaps, given in somewhat unnecessary detail, she again meets her old lover William. The latter, to atone for the wrong he once did Esther, secures a release from his unhappy marriage, and makes her his wife. He has now become a prosperous bookmaker, and the narrative of their life at a public-house of which he is the proprietor sparkles with gems of humour and realistic touches. The end is a sad one, as William, after a few years of varying fortune, dies of consumption, and Esther returns to her old mistress to end in uneventfulness an eventful life. The story is one which paints the horrors of betting, drinking, and other vices of humble life in no ambiguous colouring, but it unnecessary mud is stirred up to point a useful moral the mud soon subsides, and leaves the atmosphere purer and fresher for the treatment. The only unnatural part of the story is that which deals with the spontaneous promptings of Esther's untaught philosophy, for though her philosophy may be that of an uneducated kitchen maid who can neither read nor write, the language in which she expresses it is certainly not the language of an illiterate.

### Medburn Dumb-bell Exercises.

Mr. Farrington's new book will be welcomed by all promoters of physical drill, and those who have already found out by experience the worth of his former works will not regret the addition of this little book to their number. For the sake of others who have not yet been introduced to the Medburn series, it may be well to say that it consists of a collection of good practical guides to musical drill of all kinds, beginning at the early period of child-life when nursery rhymes stand unrivalled in infant literature. The series is published by J. Curwen and Sons, 8 and 9, Warwick Lane, E.C. Setting aside the earlier books, we turn more particularly to those which touch on "musical drill." The first treats the subject as a whole, while the next two volumes describe in detail the groups of exercises belonging to each kind of apparatus. The Medburn "Dumb-bell Exercises" is the last published, and it explains every movement with such perfect clearness and precision that it would be extremely difficult for any misunderstanding to arise. The plan of the book is as simple as could be desired, the page with directions being divided into three columns, the first containing in large clear characters the words of command corresponding to the movement which is explained more fully, in words in the centre division, and which is further illustrated in the third space by very good woodcuts. Facing each page of directions is some well-known tune to which the exercise may be easily done, and these are wisely chosen from such time-honoured airs as "The Sailor's Hornpipe" and "The British Grenadiers," each selection being so arranged that the number of bars may correspond to the right time allotted for each separate movement. For one of the "charging" exercises at the end of the book Mr. Farrington gives an appropriate and spirited fragment of his own composition. This little work is published in a neat volume, conveniently bound for the piano, and, containing some forty-six pages only, it is light to hold for reading, while the print is particularly clear both in the text and the music. Now that the importance of physical training is almost universally recognised, the question sometimes arises in schools and home circles as to what exercises are best for beginners, and also what music should be played. The serious difficulty met with by the novice and expressed in such words as "How many bars of this piece am I to play?" or "How are we to change from one tune to another?" may be entirely overcome by the study of the above-mentioned little work, and by its means many a collection of raw recruits may be formed into well-trained, smart-looking squads.

---

### JUNE MAGAZINES.

The English Illustrated Magazine is always pleasant reading. The present issue is no exception. If alone by virtue of two short stories in the June number, its reputation is well maintained. These are, "The Blue-throated God," by Flora Annie Steel, and George Girsing's "Honeymoon," both profusely illustrated. "How the Other Half Lives," by Elizabeth Banks, describes a personal exploit under the assumed profession of a flower girl, the profits at the end of a long, hard day bringing in the worthy sum of 2s. 4d.

Sunday at Home, besides much other readable matter, gives us an epitome of the work of the Young Men's Christian Association, this month celebrating its jubilee, it having been founded in St. Paul's Churchyard on June 6th, 1844. The association has thus held on its way during the greater part of the Queen's reign, and its work has so developed that there are now upwards of 5,000 associations in different parts of the world. The whole history of the society has been influenced by the circumstances of its origin. It grew out of the spiritual needs of a commercial house, and has during the half century of its existence assumed the gigantic proportions to which we have alluded.

This month's Leisure Hour contains two good scientific contributions in "The Wings of Insects," by Lewis Wright, and "Modern Hygiene in Practice," by Dr. Schofield. "The War Tax of Europe," an interesting paper on an interesting subject, reminds us of many strange facts in connection with it. Among other statistics, Mr. W. J. Gordon points out that "the Continent, including European Russia, spends £146,000,000 a year on, what it is pleased to term, purposes of defence." For this it keeps three million of men constantly under arms. On the Continent, our authority states, there are at present just four times as many soldiers proportionately as we have police. In the county of London there are 400 people to every policeman. The War Budgets of the different countries are further set forth side by side for purposes of contrast.

